# Progression-free survival 3 of 22 months achieved through third-line therapy with adebrelimab in patient with recurrent chordoma: a case report

**DOI:** 10.3389/fonc.2026.1850680

**Published:** 2026-06-29

**Authors:** Liping Sun, Juan Lang, Weiying Zhang, Zhongkui Xiong

**Affiliations:** 1 Department of Pathology, Shaoxing People’s Hospital, Shaoxing, Zhejiang, China; 2 School of Medicine, Shaoxing University, Shaoxing, Zhejiang, China; 3 Department of Radiation Oncology, Shaoxing Second Hospital, Shaoxing, Zhejiang, China

**Keywords:** adebrelimab, chordoma, immunotherapy, programmed death-1, programmed death-ligand 1, recurrence

## Abstract

Lower cervical spine origin chordomas are exceedingly uncommon tumors, and treatment may present difficulties in particular areas of the cervical spine owing to its specific anatomic architecture and the interrelationship with the vertebral arteries, cervical nerve roots, and spinal cord. Due to its rarity, the mechanisms underlying tumorigenesis and optimal therapeutic strategies for recurrence remain poorly understood. A 57-year-old Chinese female with large vertebral and paravertebral lesions from C6 to T1 underwent surgically resection on Aug 13, 2018. The pathological diagnosis in the post-operative report was chordoma close to the resection margin. No adjuvant treatment was administered after surgery. The first recurrence occurred three years postoperatively and was associated with a high tumor burden. At that time, paraplegia (lower limb paralysis and involuntary urinary and fecal control disorders) occurred. This patient received apatinib tablets, a vascular endothelial growth factor receptor (VEGFR)-tyrosine kinase inhibitor (TKI), as first-line treatment. Second-line administration of a VEGFR-TKI anlotinib capsules and programmed death-1 (PD-1) inhibitor toripalimab injection; third-line administration of the programmed death-ligand 1 (PD-L1) inhibitor adebrelimab injection, resulted in a progression-free survival 3 of 22 months and an overall survival of 7.5 years. To the best of our knowledge, this represents the first reported case of adebrelimab use in immunotherapy for chordoma.

## 1 Introduction

Chordomas are slow-growing primary malignant bony tumors that arise from the notochordal tissue in the midline of the axial skeleton (1). Approximately 300 new cases occur in the US each year, accounting for 20% of primary spinal tumors and 1%–4% of all malignant bone tumors (2). Chordomas are characterized by a high degree of invasiveness and the involvement of important neighboring structures creates major difficulties in treatment, although their growth is very slow (3). Resection with negative margins is the basic treatment principle; nonetheless, a major risk is represented by local recurrence (1) because of the lower recurrence rate in patients with en bloc resection compared to that of piecemeal or intralesional treatment (4). Chordomas present significant challenges in achieving complete gross resection, and residual tumors often inevitably progress (2, 5). The tumor is insensitive to systemic chemotherapy (6).

Although surgery remains the cornerstone of treatment, and despite advancements in techniques aimed at optimizing total tumor resection, the rate of recurrence continues to be high and the likelihood of disease-free survival (DFS) remains low (7). A retrospective analysis was conducted in a cohort of patients with chordomas affecting the mobile spine and sacrum who underwent surgical treatment. The overall survival (OS) rates at 5 and 10 years were 78% and 54%, respectively. Additionally, the local relapse-free survival (LRFS) rates at these time points were 52% and 33%, whereas the distant relapse-free survival (DRFS) rates were 86% and 72%, respectively (8). Another study reported OS rates at 5 and 10 years of 67.6% and 39.9%, respectively (2, 9). A multidisciplinary approach integrating genetics, immunotherapy, radiation therapy, and surgery at a facility with expertise in managing this complex disease provides patients with the best opportunities for survival and improved quality of life (7). Due to its rarity, the mechanisms underlying tumorigenesis and optimal therapeutic strategies for recurrence remain poorly understood (5). Targeted therapy and immunotherapy for chordomas are the latest research hotspots (5).

Chordomas typically exhibit a ‘hot’ immune microenvironment compared to that by other sarcomas (10). In total, 94.9% of chordoma samples exhibited positive programmed death-ligand 1 (PD-L1) expression in the tissue microarray. The expression score of PD-L1 in metastatic chordomas is significantly higher than that in nonmetastatic chordomas (11). The expression of PD-L1 in tumor-infiltrating lymphocytes (TILs) serves as an independent predictor of both LRFS and OS in patients with spinal chordoma (12). To date, there have been no reported cases of adebrelimab, an anti-PD-L1 antibody, being used as third-line therapy following progressive disease (PD) in patients with chordoma. Here, we present a case report of recurrent chordoma treated with third-line therapy using adebrelimab following secondary disease progression. Progression-free survival (PFS) 3 (PFS3) was reported to be 22 months, while the OS was 7.5 years.

## 2 Case presentation

A 57-year-old Chinese female patient went to Shanghai Cancer Hospital in August 2018 for the evaluation and treatment of chordoma. On August 13, 2018, surgical resection was performed to remove the large tumors in the vertebral and paravertebral regions from the sixth cervical vertebra (C6) to the first thoracic vertebra (T1). Pathology diagnosis: (C6-T1 vertebral and paravertebral lesions) clear cell tumor of 4.5 × 4.5 × 3 cm size with negative margins (local very close to the margin). This was combined with the results of light microscopy morphology and immunohistochemistry, which were consistent with chordoma. Immunohistochemical results: tumor cells: AE1/AE3 (+), EMA (+), S-100 (+), SOX-10 (-), Brachyury (+), Vimentin (+), PAX8 (-), CA9 (-), CD10 (-), Ki-67 (+, 2%) (**Supplementary Figure S1**). The patient did not undergo adjuvant therapy postoperatively. Further, on August 5, 2021, enhanced magnetic resonance imaging (MRI) showed multiple nodules and mass shadows in the neck, especially around C4, the postoperative area; the right supraclavicular region; the right thoracic entrance; and suspicious tumor recurrence. Efficacy assessment: tumor recurrence. Targeted therapy with apatinib tablets (0.25 g orally once daily) was administered as first-line treatment from September 2021. In October 2021, the patient exhibited lower limb weakness, and subsequently discontinued treatment due to intolerable adverse effects. Computed tomography (CT) scan of the neck performed in January 2022 revealed postoperative changes compatible with chordomas, multiple nodules, and masses (including the posterior margin of the fourth cervical vertebra, right cervicothoracic operation area, right supraclavicular area, and right thoracic entrance), with destruction of the right 1st rib. Besides, few slightly enlarged lymph nodes were found near the neck vessels (both sides) and the right mandible. The spinal cord signals were heterogeneous, and the lower part of the cervical and upper part of the thoracic spinal cord were slightly thinner; thus, clinical correlation was recommended. An MRI of the lumbar spine performed on the same day showed degenerative changes, mild disc bulging, and protrusion at the levels of L3/4, L4/5, and L5/S1, and mild edema of the lumbosacral subcutaneous tissues. Taking into account the patient’s medical history, the above-mentioned findings were suggestive of progression of disease (PD) in accordance with RECIST v1.1 guidelines.

**Figure 1:**
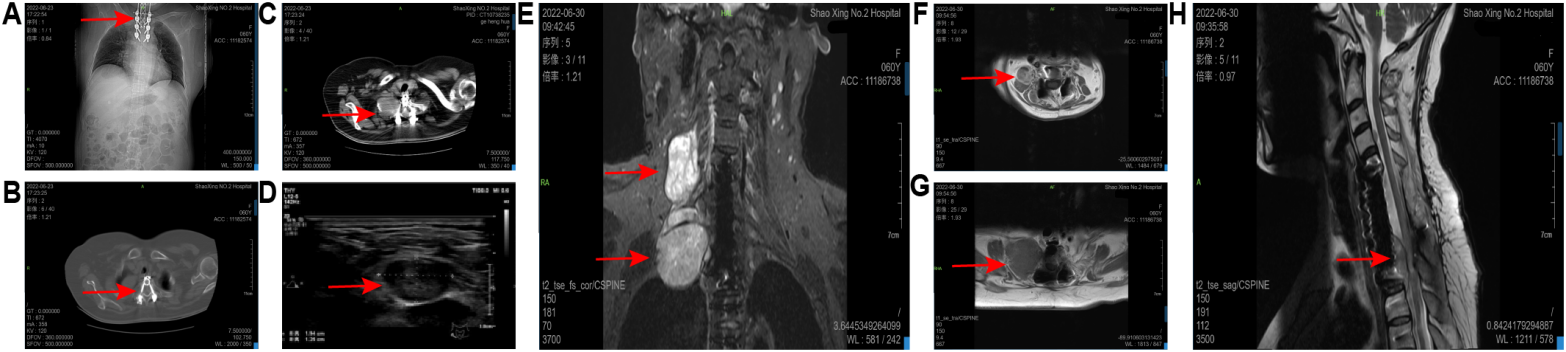
Imaging findings following the patient’s second recurrence and prior to initiating second-line therapy. **(A-C)** A chest CT scan on June 23, 2022; **(A)** Metallic shadow observed postoperatively on CT localization image; **(B)** Metallic shadow observed postoperatively on CT horizontal section image; **(C)** A soft tissue lesion located in the right cervical region, with involvement of the adjacent right first rib; **(D)** A lymph node located in the right supraclavicular region, with the largest dimension measuring approximately 19.4 × 12.5 mm, observed on an ultrasound image dated June 25, 2022; **(E-H)** An enhanced MRI of cervical spine conducted on June 30, 2022; **(E)** The lesions located on the right side of the cervical vertebrae indicated by the arrow above and on the right thoracic inlet indicated by the arrow blow exhibited long T2 signal intensities; **(F)** A lesion on the right side of the cervical vertebrae exhibited long T1 signal intensities; **(G)** A lesion on the right thoracic inlet exhibited long T1 signal intensities; **(H)** A lesion located at the posterior margin of the cervical vertebra, applying pressure on the cervical spinal cord.

**Figure 2:**
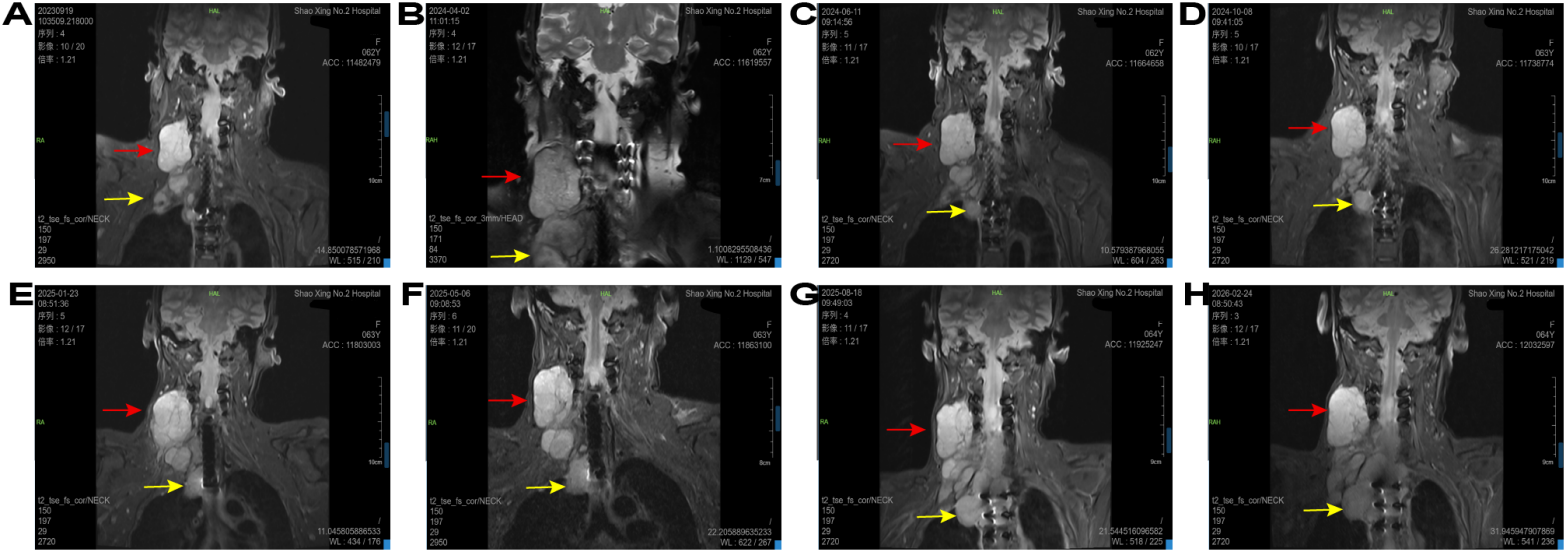
MRI findings of the patient’s cervical region from the second recurrence to the present. For the patient, the sum of the longest diameters of up to three target lesions was calculated. **(A)** Several scattered lesions identified at the right thoracic inlet on September 19, 2023, as indicated by the yellow arrow, were initially detected as a single lesion on June 30, 2022. Following second-line therapy with toripalimab in conjunction with anlotinib, the lesions subsequently fragmented into several scattered lesions; **(B)** An enhanced MR examination on April 2, 2024 revealed a notable increase in the tumor lesions when compared to prior assessments, thereby corroborating the findings from the CT scan performed the previous day. At this stage, the treatment response was evaluated as progressive disease (PD). Subsequently, third-line therapy with adebrelimab was initiated; **(C-H)** The sizes of the tumor lesions exhibited a consistent stability from June 11, 2024, to August 18, 2025. This episode occurred during the patient’s third-line treatment with adebrelimab; **(C)** MR image performed on June 11, 2024; **(D)** MR image conducted on October 8, 2014; **(E)** MR image on January 23, 2025; **(F)** MR image on May 6, 2025;**(G)** MR image on August 18, 2025; **(H)** MR image on February 24, 2026.

On June 23, 2022, the patient presented to our hospital with a chief complaint of a right-sided cervical mass, accompanied by progressive bilateral lower limb weakness and urinary and fecal incontinence. Physical examination revealed a firm, non-mobile, approximately 5-cm-diameter mass located in the right cervical region. Neurological assessment demonstrated flaccid paralysis (Medical Research Council [MRC] grade 0) in both lower limbs. The Numeric Rating Scale (NRS) for pain was 0, indicating absence of subjective pain. Chest computed tomography (CT) revealed a metallic artifact following internal fixation of the cervicothoracic vertebrae (**Figures 1A, B**). A soft tissue lesion measuring approximately 48 × 51 mm was identified in the right cervical region, with involvement of the adjacent right first rib (**Figure 1C**). On June 25, 2022, supraclavicular ultrasound examination revealed multiple lymph nodes in the right supraclavicular region, with the largest measuring approximately 19.4 × 12.5 mm and exhibiting an ill-defined cortical margin (**Figure 1D**). An enhanced MRI of cervical spine was done at our hospital on June 30, 2022, with multiple space-occupying lesions on the obliquely laterally side of C4-T1 and behind the margin of T2–4 vertebra and local bone destruction (**Figures 1E–H**), based on which it was diagnosed as PD according to the imaging results and the patient’s past history. Upon multidisciplinary discussion with the patient’s informed preferences, the patient got immunotherapy with targeted therapy, toripalimab injection 240 mg d1 plus anlotinib capsule 12 mg d1-14, 3 weeks per cycle from July 3, 2022 to September 5, 2022. On September 5, 2022, a CT scan of the neck revealed that multiple nodules and masses were identified on the right side of the fourth cervical vertebra, within the right cervicothoracic surgical region, the right supraclavicular area, and the right thoracic inlet. These lesions exhibited well-defined margins, irregular morphology, and mild heterogeneous enhancement. The largest lesion measured approximately 55 × 47 mm and demonstrated involvement of the right first rib. Efficacy assessment: stable disease (SD). The therapeutic regimen was switched to toripalimab 240 mg intravenous infusion every 3 weeks because of the patient’s intolerance to anlotinib. On September 19, 2023, an enhanced MR results indicated that multiple lesions were identified on the right side of the fourth cervical vertebra, the right cervicothoracic surgical region, the right supraclavicular area, and the right thoracic inlet. These lesions exhibited prolonged T1 and T2 signal intensities with mildly heterogeneous enhancement. The largest lesion measured approximately 56 × 42 mm and demonstrated internal cystic degeneration and necrosis indicated by the red arrow. The tumor lesion at the thoracic inlet position indicated by the yellow arrow on the MR of June 30, 2022, had dispersed into multiple small lesions (**Figure 2A**).

In March, 2024 the patient reported worsening neck pain. One month later, a chest CT scan on April 1, 2024, showed a mass on the right side of the cervicothoracic area and right thoracic entrance, with destruction of the right first rib. The mass increased in size compared to that in the previous examination on September 17, 2023. An enhanced MR examination on April 2, 2024, indicated that multiple masses were identified on the right side of the fourth cervical vertebra, the right cervicothoracic junction, the right supraclavicular region, and the right thoracic inlet. These lesions exhibited long T1 and long T2 signal intensities, with mildly heterogeneous enhancement. The largest lesion measured approximately 56 × 44 mm and demonstrated cystic necrosis, rim enhancement on contrast-enhanced imaging, and restricted diffusion. The abnormalities were found to involve the right first rib and scapula (**Figure 2B**). A response assessment indicating the presence of PD was performed at this time point. Considering the progression of the patient’s disease and after excluding any contraindications, an intravenous infusion of adebrelimab at a dosage of 1.2 g was administered every 3 weeks, starting from April 5, 2024 to the present date. During the subsequent follow-up period, the MR examination results indicated that the lesion remained stable from June 11, 2024 to August 18, 2025, (**Figures 2C–G**) and progressed on MR images conducted on February 24, 2026 (**Figure 2H**).

**Figure 3:**
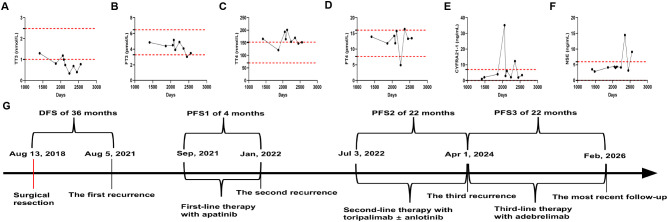
Laboratory test results and timeline. **(A-G)** The horizontal axis denotes time, with the date of the initial surgical pathological report (August 13, 2018) designated as day 0; **(A)** TT3 levels; **(B)** FT3 levels; **(C)** TT4 levels; **(D)** FT4 levels; **(E)** CYFRA21-1; **(F)** NSE; **(G)** The timeline of the course outlining the patient’s treatments and their effectiveness. (i) The patient underwent surgical resection on August 13, 2018. Histopathological examination, finalized on August 28, 2018, confirmed a diagnosis of chordoma. No adjuvant therapy was administered. The first recurrence was identified via MRI on August 5, 2021, and the patient’s DFS duration was 36 months. (ii) Second-line treatment with apatinib was initiated in September 2021, but subsequently discontinued in December 2021 due to intolerable adverse effects. The patient experienced the second disease recurrence in January 2022, resulting in PFS1 of 4 months. (iii) The patient declined radiotherapy and chemotherapy. On July 3, 2022, second-line treatment with toripalimab in combination with anlotinib was initiated. However, anlotinib was subsequently discontinued due to intolerable adverse events. Toripalimab monotherapy was continued. A MRI scan performed on April 1, 2024, revealed the patient’s third disease recurrence, resulting in PFS2 duration of 22 months. Subsequently, the patient received third-line treatment with adebrelimab. Under this regimen, the treatment response was stable disease (SD). The most recent follow-up occurred in February 2026, and PFS3 duration was also 22 months.

The changes in the laboratory test items of patients during the process of immunotherapy combined with or without targeted therapy were as follows. The thyroid hormone test results, illustrated in **Figures 3A-D**; **Supplementary Table S1**, were observed following immunotherapy. On October 1, 2024, the lowest recorded value of TT3 was 0.34 nmol/L; on April 28, 2025, the minimum value of FT3 reached 4.28 pmol/L; on June 7, 2024, the highest recorded value of TT4 was noted at 201.43 nmol/L; and on October 1, 2024, the lowest level of FT4 measured was found to be 4.91 pmol/L The patient is receiving hormone replacement therapy with levothyroxine. The highest value of CYFRA21–1 was 35.17 ng/ml, measured on April 1, 2024. Imaging examinations indicated tumor progression, and the treatment plan was subsequently adjusted to third-line treatment with adebrelimab, as shown in **Figure 3E**. The peak NSE level was 14.47 ng/mL, recorded on January 23, 2025. No adjustments were made to the treatment regimen at that time, and the value subsequently declined to within the normal reference range, as shown in **Figure 3F**. According to the Common Terminology Criteria for Adverse Events (CTCAE) v5.0, the adverse reactions related to drug treatment were assessed, and the result showed grade 2 hypothyroidism, and grade 3 anemia. The patient received ferrous succinate tablets for the treatment of anemia. The patient’s diagnosis and treatment process is shown in **Figure 3G**; **Supplementary Table S2**.

## 3 Discussion

Lower cervical spine chordomas are extremely rare neoplasms (13, 14). Treatment choice is difficult at specific locations, particularly the cervical spine, owing to its anatomy and intimate relationship with the vertebral arteries, cervical nerve roots, and spinal cord (15). In this case, for the chordoma which was located in the lower cervical spine, the postoperative pathological report indicated that the surgical margins were negative but close. As recommended in the NCCN Clinical Practice Guidelines in Oncology (Bone Cancer, Version 1.2020), radiotherapy is not indicated following R0 resection of chordoma. For local recurrence, radiotherapy is recommended if the lesion is unresectable (Category 2B). However, in accordance with the ESMO–EURACAN–GENTURIS–ERNPaedCan Clinical Practice Guideline for Bone Sarcomas (v2021), adjuvant radiotherapy should routinely be considered for patients with cervical spine chordoma (16). There remains ongoing debate concerning the necessity of postoperative radiotherapy, with significant variability in recommendations across major clinical practice guidelines. The tumor’s intrinsic radioresistance renders radiation therapy clinically challenging (17). Among currently available radiotherapeutic techniques, pencil-beam scanning intensity-modulated proton therapy and carbon ion therapy represent the most promising approaches for managing these neoplasms (17). Pencil beam scanning proton therapy for chordomas and chondrosarcomas yielded 7-year OS, local control (LC), and distant control (DC) rates of 86.3%, 87.7%, and 95.7%, respectively (18). A meta-analysis demonstrated that proton therapy is a clinically effective local treatment strategy for chordomas, offering a favorable safety profile. The LC rates at 1, 2, 3, 5, and 7 years across these studies were 97%, 92%, 88%, 77%, and 71%, respectively. The corresponding OS rates at 1, 2, 3, 5, and 7 years were 100%, 90%, 89%, 86%, and 80%, respectively (19).

As recommended by the NCCN Clinical Practice Guidelines in Oncology (Version 1.2026) for Soft Tissue Sarcoma (STS), anthracycline-based regimens are the preferred first-line therapy and subsequent lines of therapy for advanced/metastatic STSs. Chordoma represents a distinct subtype within the broader category of STSs, which encompass a heterogeneous group of malignant mesenchymal tumors (20). Chordoma exhibits resistance to conventional chemotherapy and radiotherapy (21). No FDA-approved effective systemic therapies are currently available for chordomas (22, 23). Chordoma is characterized by a low tumor mutational burden (24), which constrain the utility of genomic approaches in therapeutic target discovery (25). The most distinctive genomic alterations in chordoma are homozygous deletions of the cyclin-dependent kinase inhibitor 2A/B (CDKN2A/B) and inactivating mutations or deletions of (33%), low-density lipoprotein receptor-related protein 1B (LRP1B) (12%), polybromo-1 (PBRM1) (11%), and epidermal growth factor receptor (EGFR) (9%) (24).Within 3 years of disease onset, the tumor recurred. At that time, the patient presented with symptoms of paraplegia, including loss of motor power in the lower extremities and impaired voluntary control of urination and defecation. A survey on the management of spinal chordomas was distributed electronically to members of the AOSpine Knowledge Forum Tumor. The most controversial topic is the indication for postoperative radiotherapy. In particular, 41% of the respondents advocated using radiotherapy when only positive margins were found and 38% when margin status did not matter (26). The inherently slow growth rate of the tumor, together with the tumor margins’ postoperative status, are crucial determinants of patient survival. Local recurrence is an unfavorable feature in chordomas, as the neoplasm’s metastatic characteristic is not common despite being reported after incomplete resection (13). Due to their locally aggressive biology and invasiveness, chordomas can lead to catastrophic consequences, with median OS (mOS) of 10 years after diagnosis (7). Given the long survival rate with non-surgical management, chordomas are thought to be relatively indolent (4).

During treatment, apatinib was used as a first-line therapeutic agent and achieved a PFS1 of only 4 months. Here, PFS1 denotes the time interval from the initiation of first-line therapy to disease progression (27). Owing to the tolerability profile of the drug, the patient was unable to undergo long-term targeted treatment. Apatinib is a small-molecule tyrosine kinase inhibitor (TKI) that specifically targets vascular endothelial growth factor receptor-2 (VEGFR-2), thereby effectively inhibiting tumor angiogenesis (28). It is also a potent TKI of platelet-derived growth factor receptor-β (PDGFR-β), rearranged during transfection (RET), c-Src, and c-Kit (29). Both VEGFR1 and VEGFR2 were robustly expressed not only on vascular endothelial cells but also on tumor cells in a chordoma cell line and in 54 chordoma tissue specimens (30). In a single-arm, single-center phase 2 study evaluating apatinib in patients with advanced chordoma, the medium PFS (mPFS) was 18 months (31). Pazopanib is a small molecule inhibitor of VEGFR1-3 (32). Pazopanib was approved by the China National Medical Products Administration (NMPA) in 2017 for first-line treatment of advanced renal cell carcinoma (RCC) as well as for the treatment of advanced RCC in patients previously treated with cytokine-based therapy. mPFS was 4.6 months with pazopanib versus 1.6 months with placebo in a randomized, double-blind, placebo-controlled phase 3 trial in patients with metastatic STSs (PALETTE) (33).

The patient received toripalimab plus anlotinib as a second-line therapy regimen, yielding a markedly prolonged PFS2 of 22 months. PFS2 refers to the time interval from the initiation of second-line therapy to disease progression. The expression of PD-L1 was detected in 86.0% of 92 tissue specimens (34). Dridi et al. stained 81 chordoma specimens and discovered that PD-L1 was present in inflammatory cells but not in tumor cells (35). In a separate study conducted using immunofluorescence staining of 92 chordomas, PD-L1 expression was detected in the tumor cells of some patients (36). In another report, PD-L1 staining was sparse in both tumor and immune cells, with scores <1 observed in 38 of 41 patients (92.7%) and 22 of 25 patients (88%), respectively (37). PD-L1 positive immune cells in patients with chordoma tumors appear to have a negative prognosis (35). The density of programmed death-1 (PD-1) (+) and PD-L1 (+) TILs, both within the tumor immune microenvironment and in the combined tumor regions (including the TI and invasion margin), demonstrated a significant association with LRFS and OS (38). The PD-L1 (+) chordoma phenotype is significantly correlated with advanced disease stage and poor LRFS (39). The co-expression of HHLA2 and PD-L1 in tumor cells independently predicted poorer LRFS and OS outcomes (34). An analysis of 1,407 ultra-rare sarcomas from the AACR GENIE database revealed that tumor mutational burden (TMB) was generally low across all four sarcoma subtypes; however, substantial inter-subtype heterogeneity was observed—specifically, 3.8% of soft-tissue ultra-rare sarcomas (ST URS) and 0.55% of bone ultra-rare sarcomas (bone URS) exhibited high TMB (40). A mutational analysis of 86 primary and advanced tumor samples from chordoma patients, performed using next-generation sequencing, revealed that microsatellite instability was not detected in 7 of 69 samples (10.1%) (37).

Immunotherapy for chordoma represents a promising and increasingly active area of investigation, though clinical evidence remains limited to a small number early-phase clinical trials (41). Several clinical studies investigating PD-(L)1–targeted immunotherapy for chordomas have demonstrated its measurable clinical efficacy. In a systematic literature review of chordoma clinical trials, PD-1 inhibitors were the most frequently employed therapeutic agents, featured in 50.0% of the trials; PD-L1 inhibitors ranked second (36.4%), followed by CTLA-4 inhibitors (22.7%) and mTOR inhibitors (13.6%). Phase II studies were the most prevalent (40.9%), followed by Phase I–II trials (31.8%) and Phase I trials (27.3%) across the 22 clinical trials included in the analysis (42). Avelumab may boost endogenous NK cell activation as a monotherapy for chordoma and could potentially stimulate the cytotoxic activity of NK cells against chordoma cells by antibody-dependent cellular cytotoxicity (ADCC), when administered with T-cell immunotherapy, such as the chordoma vaccine (2). A retrospective study involved 17 patients diagnosed with recurrent chordomas who received immune checkpoint inhibitors (ICIs) as part of their treatment for progressive disease. Most patients were treated with pembrolizumab (n=9, 53%), and the median number of cycles administered was eight. The 1-year OS rate was 87% while the 1-year PFS was 56%, with mPFS of 14 months. Following the initiation of ICIs therapy, most patients (n=15, 88%) experienced clinical benefits, including complete response (CR; 6%), partial response (PR; 18%), and stable disease (SD; 65%). Among responders (n=15), the median duration of response (DOR) was 12 months (43). An analysis of a non-randomized, open-label, phase 2 basket trial evaluating pembrolizumab in patients with rare and ultra-rare sarcomas (AcSé Pembrolizumab) revealed that 97 individuals received treatment and were included in the analyses. Among these, 34 (35%) had chordomas, with a median follow-up period of 13.1 months. At week 12, the objective response rate (ORR) was 6.2%, which included no CRs and six PRs (44). A single-center phase 2 trial investigating the combination of durvalumab and tremelimumab in patients with advanced or metastatic soft tissue and bone sarcomas enrolled 62 participants, including five patients with chordoma. With a median follow-up period of 37.2 months, the PFS rate at 12 weeks was observed to be 49% (45). A Phase II trial demonstrated that the combination of durvalumab and pazopanib exhibited promising efficacy in an unselected cohort of patients with STSs, with mPFS of 7.7 months and a manageable toxicity profile (46). In a phase 2 study of atezolizumab in patients with advanced alveolar soft-part sarcoma (ASPS), PD-L1–negative patients were not excluded. This design choice reflects the growing recognition that patient selection based solely on pretreatment PD-1 or PD-L1 expression status may inadvertently exclude individuals with ASPS who could still derive meaningful clinical benefit from immune checkpoint inhibitor therapy. Tumor immunogenicity, more specifically, the capacity to acquire an immunotherapy-responsive phenotype, is dynamic and can evolve during treatment (47). Indeed, adaptive upregulation of PD-L1 expression in tumor cells has been well documented in melanoma, where on-treatment (rather than baseline) PD-L1 expression in tumor tissue demonstrates superior predictive value for response to immune checkpoint inhibitors (48). The phase II clinical study of nivolumab and relatlimab in treating participants with advanced chordoma is currently under trial enrollment (49).

A phase I trial was conducted to evaluate the efficacy of toripalimab in patients with 33 advanced or recurrent malignancies. This cohort included 12 patients diagnosed with ASPS, seven with non-small-cell lung cancer, and 11 with lymphoma. The mOS for patients with ASPS was found to be 34.7 months (50). Toripalimab, a humanized IgG4 monoclonal antibody that specifically targets human PD-1, has been approved by the U.S. Food and Drug Administration (FDA) for the treatment of nasopharyngeal carcinoma (51). This observational study was conducted to evaluate the efficacy and safety of toripalimab in combination with doxorubicin as a first-line treatment for metastatic STSs. The ORR was 36.7%, and the disease control rate was 80%. The mPFS was recorded at 8 months. The most frequently reported adverse events were nausea (66.7%), fatigue (60%), and vomiting (40%) (52).

Anlotinib is a TKI that targets multiple receptors, including VEGFR 1–3, PDGFR-α/β, fibroblast growth factor receptors (FGFR) 1–4, c-Kit, c-FMS, and discoidin domain receptor 1 (DDR1) (29). It demonstrates significant potential for both antineoplastic and antiangiogenic effects (53). A retrospective, multi-institutional study evaluated the efficacy of anlotinib in patients with unresectable or metastatic bone sarcomas. A total of 48 patients were analyzed, comprising 27 with osteosarcoma, nine with chondrosarcoma, eight with Ewing’s sarcoma, and three with chordoma. The overall response rate among all patients was 10.4%, and the mPFS was recorded at 4.6 months. Furthermore, the PFS rates at 3 and 6 months were 72.9% and 35.4%, respectively (54).

It is well established that angiogenesis constitutes a critical process in tumorigenesis, playing a pivotal role in both primary tumor growth and metastatic dissemination (55). A network meta-analysis demonstrated that the short-term efficacy of small-molecule anti-angiogenic TKIs varied across bone and STSs, with trends favoring the combination of apatinib and chemotherapy as well as anlotinib monotherapy (56). Vascular endothelial growth factor (VEGF) has been implicated in promoting immune evasion (57). Tumor cells or M2 macrophages were capable of transducing VEGF signaling to upregulate PD-L1 expression (58). Inhibiting VEGF signaling may promote normalization of the tumor vasculature, thereby enhancing immune cell infiltration (59). Both apatinib and sunitinib inhibit VEGFR2, thereby suppressing PD-L1 expression in osteosarcoma through STAT3 targeting (60, 61). In PD-L1^low^ and immune desert–like mouse tumors, anti–VEGF therapy promoted increased infiltration of CD8^+^ T cells (62). Anti-VEGF therapy enhances immune checkpoint blockade efficacy via B cell activating factor- and interleukin-12-dependent reprogramming of the tumor microenvironment in a murine model (63). Recent biological research provides mechanistic support for the current clinically successful therapeutic strategy of dual blockade of VEGF and PD-1 signaling in cancer treatment (58).

Disease progression was observed following second-line therapy, after which the patient received adebrelimab as third-line treatment, resulting in a substantial PFS3 of 22 months. PFS3 denotes the time interval from the initiation of third-line therapy to disease progression. Adebrelimab is a recombinant humanized immunoglobulin G4 antibody that exhibits strong affinity for both human and monkey PD-L1 antigens, while demonstrating negligible binding to the B7 DC molecule and B7-H3 (64). A multicenter, randomized, double-blind, placebo-controlled phase 3 trial (CAPSTONE-1) demonstrated that the addition of adebrelimab to chemotherapy with carboplatin and etoposide significantly improved OS while maintaining an acceptable safety profile in patients with extensive-stage small cell lung cancer (ES-SCLC) (65). Based on the findings of this clinical trial, adebrelimab was approved by NMPA in China as a first-line therapy in combination with carboplatin and etoposide in adult patients diagnosed with ES-SCLC (66). Survival analysis based on reconstructed patient-level data demonstrated that treatment with adebrelimab significantly prolonged survival of ES-SCLC compared to that with treatment with atezolizumab or durvalumab (67). To date, there have been no reports on the use of adebrelimab for the treatment of chordoma. In this patient, the administration of adebrelimab as third-line treatment resulted in stable disease.

The patient in this case study remains alive despite experiencing three relapses and having received three lines of therapy: first-line treatment consisted of apatinib monotherapy whereas second-line treatment combined toripalimab with anlotinib and third-line therapy with adebrelimab. However, this case report has certain limitations that should be acknowledged. (a) Postoperative pathological results were available; however, corresponding pathological images were not provided, and the patient declined to undergo additional biopsy. (b) Information regarding immunotherapy-related biomarkers, such as PD-L1 expression, microsatellite instability status and tumor mutational burden was not provided. (c) The patient did not receive postoperative radiotherapy or subsequent proton or heavy ion therapy following disease recurrence.

Following a comprehensive review of this case, the following key points were identified: (a) multidisciplinary discussion plays a critical role in formulating treatment strategies for rare tumors, such as chordoma. (b) A robust family support system is essential to ensure patients’ quality of life during outpatient care. (c) Despite the development of lower limb paralysis and progressive loss of urinary and bowel control following the first recurrence, both treatment regimens demonstrated clinical efficacy, with the patient remaining alive as of February 2026. These outcomes suggest that immunotherapy and targeted therapy represent important systemic approaches for managing such cases. However, this requires confirmation through higher-level, evidence-based medical research. The exceptionally low incidence of chordoma poses substantial challenges to the initiation of high-quality, large-scale clinical trials. The establishment and enhancement of a global case repository for rare diseases represents a viable and strategically valuable approach.

## Data Availability

The raw data supporting the conclusions of this article will be made available by the authors, without undue reservation.

## Supplementary material

Supplementary Figure S1 Postoperative pathological report.

Supplementary Table S1 Changes in laboratory test parameters during second-line and third-line therapies.

Supplementary Table S2 Efficacy assessment based on RECIST 1.1 criteria in second-line and third-line treatments.

## References

[1] Wang K Osei-Hwedieh DO Walhart TA Hung YP Wang Y Cattaneo G . B7-H3 CAR-T cell therapy combined with irradiation is effective in targeting bulk and radiation-resistant chordoma cancer cells. J Immunother Cancer. (2025) 13:1–13. doi: 10.1136/jitc-2024-009544 39848690 PMC11784168

[2] Fujii R Friedman ER Richards J Tsang KY Heery CR Schlom J . Enhanced killing of chordoma cells by antibody-dependent cell-mediated cytotoxicity employing the novel anti-PD-L1 antibody avelumab. Oncotarget. (2016) 7:33498–511. doi: 10.18632/oncotarget.9256 27172898 PMC5085098

[3] Baluszek S Kober P Rusetska N Wagrodzki M Mandat T Kunicki J . DNA methylation, combined with RNA sequencing, provide novel insight into molecular classification of chordomas and their microenvironment. Acta Neuropathol Commun. (2023) 11:113. doi: 10.1186/s40478-023-01610-0 37434245 PMC10337070

[4] D'Amore T Boyce B Mesfin A . Chordoma of the mobile spine and sacrum: clinical management and prognosis. J Spine Surg. (2018) 4:546–52. doi: 10.21037/jss.2018.07.09 30547117 PMC6261773

[5] Gao J Huang R Yin H Song D Meng T . Research hotspots and trends of chordoma: a bibliometric analysis. Front Oncol. (2022) 12:946597. doi: 10.3389/fonc.2022.946597 36185236 PMC9523362

[6] Gray SW Singhabhandhu B Smith RA Skandalakis JE . Sacrococcygeal chordoma: report of a case and review of the literature. Surgery. (1975) 78:573–82 1188599

[7] Connors SW Aoun SG Shi C Peinado-Reyes V Hall K Bagley CA . Recent advances in understanding and managing chordomas: an update. F1000Research. (2020) 9:1–7. doi: 10.12688/f1000research.22440.1 32724558 PMC7366033

[8] Stacchiotti S Casali PG Lo Vullo S Mariani L Palassini E Mercuri M . Chordoma of the mobile spine and sacrum: a retrospective analysis of a series of patients surgically treated at two referral centers. Ann Surg Oncol. (2010) 17:211–9. doi: 10.1245/s10434-009-0740-x 19847568

[9] McMaster ML Goldstein AM Bromley CM Ishibe N Parry DM . Chordoma: incidence and survival patterns in the United States, 1973-1995. Cancer Causes Control CCC. (2001) 12:1–11. doi: 10.1023/a:1008947301735 11227920

[10] van Oost S Meijer DM Ijsselsteijn ME Roelands JP van den Akker B van der Breggen R . Multimodal profiling of chordoma immunity reveals distinct immune contextures. J Immunother Cancer. (2024) 12:1–15. doi: 10.1136/jitc-2023-008138 38272563 PMC10824073

[11] Feng Y Shen J Gao Y Liao Y Cote G Choy E . Expression of programmed cell death ligand 1 (PD-L1) and prevalence of tumor-infiltrating lymphocytes (TILs) in chordoma. Oncotarget. (2015) 6:11139–49. doi: 10.18632/oncotarget.3576 25871477 PMC4484445

[12] Zou MX Peng AB Lv GH Wang XB Li J She XL . Expression of programmed death-1 ligand (PD-L1) in tumor-infiltrating lymphocytes is associated with favorable spinal chordoma prognosis. Am J Trans Res. (2016) 8:3274–87 27508049 PMC4969465

[13] Muenkaew Y Jinawath A Cheewaruangroj W . Lower cervical chordomas: a case report and differential diagnosis. Cancer Rep. (2025) 8:e70270. doi: 10.1002/cnr2.70270 40631598 PMC12238892

[14] Stacchiotti S Pantziarka P Leonard H Voltz C Abatedaga L Bouche G . How to foster new treatment development in ultra-rare tumours? Joint EMA-EORTC multi-stakeholder workshops on ultra-rare sarcomas as a model for rare cancers. Cancer Treat Rev. (2025) 140:103003. doi: 10.1016/j.ctrv.2025.103003 40789252

[15] Onoki T Hashimoto K Takahashi K Yahata KI Kusakabe J Kawaharada T . Resection of C2 chordoma after failed ion-beam radiotherapy: illustrative case. J Neurosurg Case Lessons. (2025) 10:1–5. doi: 10.3171/case25241 40720900 PMC12305354

[16] Strauss SJ Frezza AM Abecassis N Bajpai J Bauer S Biagini R . Bone sarcomas: ESMO-EURACAN-GENTURIS-ERN PaedCan Clinical Practice Guideline for diagnosis, treatment and follow-up. Ann Oncol Off J Eur Soc For Med Oncol. (2021) 32:1520–36. doi: 10.1016/j.annonc.2021.08.1995 34500044

[17] Lemaeva AA Gulidov IA . Radiation therapy for chordomas and chondrosarcomas of the skull base: evaluation of the effectiveness of treatment methods (review). Sovremennye Tekhnologii v Meditsine. (2023) 15:44–52. doi: 10.17691/stm2023.15.5.05 39967912 PMC11832067

[18] Vazquez M Cherchik A de Angelis C Pica A Calaminus G Weber DC . Long-term clinical outcome and quality of life of children, adolescents, and young adults with chordoma or chondrosarcoma treated with pencil beam scanning proton therapy. Pediatr Blood Cancer. (2025) 72:e31898. doi: 10.1002/pbc.31898 40682267

[19] Dong M Liu Y Bai J Li X Wu R . Proton beam therapy for chordoma: a systematic review and meta-analysis. Crit Rev Oncology/Hematology. (2026) 217:105013. doi: 10.1016/j.critrevonc.2025.105013 41223980

[20] Liu W Jiang Q Zhou Y . Advances of systemic treatment for adult soft-tissue sarcoma. Chin Clin Oncol. (2018) 7:42. doi: 10.21037/cco.2018.08.02 30173532

[21] Xu J Shi Q Wang B Ji T Guo W Ren T . The role of tumor immune microenvironment in chordoma: promising immunotherapy strategies. Front Immunol. (2023) 14:1257254. doi: 10.3389/fimmu.2023.1257254 37720221 PMC10502727

[22] Arrieta VA Benotmane JK Du R Habashy KJ Zhao J Najem H . Integrated immune profiling of chordomas reveals spatially organized niches and functional heterogeneity. Neuro-Oncology. (2025) 28:440–53. doi: 10.1093/neuonc/noaf213 41586579 PMC12979039

[23] Navarro-Garcia de Llano JP Iyer HG Hoffman HC Seetharam M Attia S Akinduro OO . Comparative outcomes of brachyury vaccine vs. imatinib in advanced chordoma: a Mayo Clinic experience. Cancers. (2025) 17:1–12. doi: 10.3390/cancers17213493 41228286 PMC12609630

[24] Zhang H Xing J Yuan Z Zhao C Yang C . Targeting the tumor immune microenvironment in chordoma: from mechanistic insights to therapeutic breakthroughs. Biochim Biophys Acta Rev Cancer. (2025) 1881:189520. doi: 10.1016/j.bbcan.2025.189520 41448553

[25] Zeinali M Seraj FQM Rawson C Azab M Karsy M . Systematic review of novel target therapies and clinical trials in chordoma. Clin Neurol Neurosurg. (2025) 259:109222. doi: 10.1016/j.clineuro.2025.109222 41177143

[26] Dea N Fisher CG Reynolds JJ Schwab JH Rhines LD Gokaslan ZL . Current treatment strategy for newly diagnosed chordoma of the mobile spine and sacrum: results of an international survey. J Neurosurg Spine. (2019) 30:119–25. doi: 10.3171/2018.6.spine18362 30497218

[27] Libert L Abdeddaim C Saleh K Even C Duplomb S Dubreuil J . Retrospective multicentric survival analysis of patients receiving TPEx regimen as first-line treatment of recurrent and/or metastatic head and neck squamous cell carcinoma. ESMO Open. (2025) 10:104544. doi: 10.1016/j.esmoop.2025.104544 40220661 PMC12017985

[28] Huang J Peng J Zhai E Wei R Qian C Li J . Clinical efficacy and safety of apatinib combined with irinotecan in HER2-negative patients with advanced gastric or gastroesophageal junction adenocarcinoma after first-line treatment failure: a single-arm, single-center retrospective study. J Gastrointestinal Cancer. (2025) 56:137. doi: 10.1007/s12029-025-01259-z 40524072 PMC12170724

[29] Deng M Zha J Jiang Z Jia X Shi Y Li P . Apatinib exhibits anti-leukemia activity in preclinical models of acute lymphoblastic leukemia. J Transl Med. (2018) 16:47. doi: 10.1186/s12967-018-1421-y 29490645 PMC5831852

[30] Morimoto Y Tamura R Ohara K Kosugi K Oishi Y Kuranari Y . Prognostic significance of VEGF receptors expression on the tumor cells in skull base chordoma. J Neuro-Oncol. (2019) 144:65–77. doi: 10.1007/s11060-019-03221-z 31240525

[31] Liu C Jia Q Wei H Yang X Liu T Zhao J . Apatinib in patients with advanced chordoma: a single-arm, single-centre, phase 2 study. Lancet Oncol. (2020) 21:1244–52. doi: 10.1016/s1470-2045(20)30466-6 32888455

[32] Li W Feng C Di W Hong S Chen H Ejaz M . Clinical use of vascular endothelial growth factor receptor inhibitors for the treatment of renal cell carcinoma. Eur J Med Chem. (2020) 200:112482. doi: 10.1016/j.ejmech.2020.112482 32492594

[33] van der Graaf WT Blay JY Chawla SP Kim DW Bui-Nguyen B Casali PG . Pazopanib for metastatic soft-tissue sarcoma (PALETTE): a randomised, double-blind, placebo-controlled phase 3 trial. Lancet. (2012) 379:1879–86. doi: 10.1016/s0140-6736(12)60651-5 22595799

[34] Xia C Huang W Chen YL Fu HB Tang M Zhang TL . Coexpression of HHLA2 and PD-L1 on tumor cells independently predicts the survival of spinal chordoma patients. Front Immunol. (2021) 12:797407. doi: 10.3389/fimmu.2021.797407 35145510 PMC8824251

[35] Dridi M Krebs-Drouot L Meyronet D Dumollard JM Vassal F Jouanneau E . The immune microenvironment of chordomas: an immunohistochemical analysis. Cancers. (2021) 13:1–20. doi: 10.3390/cancers13133335 34283048 PMC8268246

[36] Duan W Zhang B Li X Chen W Jia S Xin Z . Single-cell transcriptome profiling reveals intra-tumoral heterogeneity in human chordomas. Cancer Immunology Immunotherapy CII. (2022) 71:2185–95. doi: 10.1007/s00262-022-03152-1 35084549 PMC10992276

[37] Chan J Kendal JK Duan Z Ferreira A Samiei A Nelson SD . Mutational analysis of primary and advanced chordoma tissue using next-generation sequencing. Cancer. (2025) 131:e70033. doi: 10.1002/cncr.70033 40802536 PMC12348310

[38] Zou MX Lv GH Wang XB Huang W Li J Jiang Y . Clinical impact of the immune microenvironment in spinal chordoma: immunoscore as an independent favorable prognostic factor. Neurosurgery. (2019) 84:E318–33. doi: 10.1093/neuros/nyy274 30032257

[39] Zou MX Guo KM Lv GH Huang W Li J Wang XB . Clinicopathologic implications of CD8(+)/Foxp3(+) ratio and miR-574-3p/PD-L1 axis in spinal chordoma patients. Cancer Immunology Immunotherapy CII. (2018) 67:209–24. doi: 10.1007/s00262-017-2080-1 29051990 PMC11028121

[40] Denu RA Moyers JT Gouda MA Conley AP Lazar AJ Subbiah V . The landscape of alterations from 1407 ultra-rare sarcomas from the AACR GENIE database: clinical implications. Clin Cancer Res Off J Am Assoc For Cancer Res. (2023) 29:4669–78. doi: 10.1158/1078-0432.ccr-23-0876 37643131 PMC11874058

[41] Alexander AY Dhawan S Venteicher AS . Role of immunotherapy in treatment refractory chordomas: review of current evidence. Front Surg. (2024) 11:1375567. doi: 10.3389/fsurg.2024.1375567 38881706 PMC11177759

[42] Agosti E Antonietti S Zeppieri M Ius T Fiorindi A Tel A . Chordoma genetic aberrations and targeted therapies panorama: a systematic literature review. J Clin Med. (2024) 13:1–18. doi: 10.3390/jcm13092711 38731241 PMC11084907

[43] Bishop AJ Amini B Lin H Raza SM Patel S Grosshans DR . Immune checkpoint inhibitors have clinical activity in patients with recurrent chordoma. J Immunotherapy. (2022) 45:374–8. doi: 10.1097/cji.0000000000000431 35943386 PMC9452485

[44] Blay JY Chevret S Le Cesne A Brahmi M Penel N Cousin S . Pembrolizumab in patients with rare and ultra-rare sarcomas (AcSe Pembrolizumab): analysis of a subgroup from a non-randomised, open-label, phase 2, basket trial. Lancet Oncol. (2023) 24:892–902. doi: 10.1016/s1470-2045(23)00282-6 37429302

[45] Somaiah N Conley AP Parra ER Lin H Amini B Solis Soto L . Durvalumab plus tremelimumab in advanced or metastatic soft tissue and bone sarcomas: a single-centre phase 2 trial. Lancet Oncol. (2022) 23:1156–66. doi: 10.1016/s1470-2045(22)00392-8 35934010

[46] Cho HJ Yun KH Shin SJ Lee YH Kim SH Baek W . Durvalumab plus pazopanib combination in patients with advanced soft tissue sarcomas: a phase II trial. Nat Commun. (2024) 15:685. doi: 10.1038/s41467-024-44875-2 38263321 PMC10806253

[47] Chen AP Sharon E O'Sullivan-Coyne G Moore N Foster JC Hu JS . Atezolizumab for advanced alveolar soft part sarcoma. N Engl J Med. (2023) 389:911–21. doi: 10.1056/nejmoa2303383 37672694 PMC10729808

[48] Chen PL Roh W Reuben A Cooper ZA Spencer CN Prieto PA . Analysis of immune signatures in longitudinal tumor samples yields insight into biomarkers of response and mechanisms of resistance to immune checkpoint blockade. Cancer Discov. (2016) 6:827–37. doi: 10.1158/2159-8290.cd-15-1545 27301722 PMC5082984

[49] Traylor JI Pernik MN Plitt AR Lim M Garzon-Muvdi T . Immunotherapy for chordoma and chondrosarcoma: current evidence. Cancers. (2021) 13:1–15. doi: 10.3390/cancers13102408 34067530 PMC8156915

[50] Yang J Dong L Yang S Han X Han Y Jiang S . Safety and clinical efficacy of toripalimab, a PD-1 mAb, in patients with advanced or recurrent Malignancies in a phase I study. Eur J Cancer. (2020) 130:182–92. doi: 10.1016/j.ejca.2020.01.028 32224416

[51] Shu P Li X Zhou Q Li G Zhang K Yuan L . Multicenter phase 1/2 study of onatasertib, a dual TORC1/2 inhibitor, combined with the PD-1 antibody toripalimab in advanced solid tumors. Signal Transduction Targeted Ther. (2025) 10:198. doi: 10.1038/s41392-025-02281-0 40555721 PMC12187923

[52] Liu Z Liu C Yao W Gao S Wang J Zhang P . Efficacy and safety of toripalimab combined with doxorubicin as first-line treatment for metastatic soft tissue sarcomas: an observational study. Anti-Cancer Drugs. (2021) 32:962–8. doi: 10.1097/cad.0000000000001088 34001702 PMC8448405

[53] Chang J Xu B Li Z Wei Z Che X Cai J . Efficacy and safety of anlotinib monotherapy for advanced hepatocellular carcinoma and clinical role of alpha-fetoprotein. Sci Rep. (2025) 15:29209. doi: 10.1038/s41598-025-14759-6 40783436 PMC12335444

[54] Liu Z Gao S Zhu L Wang J Zhang P Li P . Efficacy and safety of anlotinib in patients with unresectable or metastatic bone sarcoma: a retrospective multiple institution study. Cancer Med. (2021) 10:7593–600. doi: 10.1002/cam4.4286 34564939 PMC8559478

[55] Hanahan D Weinberg RA . Hallmarks of cancer: the next generation. Cell. (2011) 144:646–74. doi: 10.1016/j.cell.2011.02.013 21376230

[56] Zhang J Liu Y Zhao X Xu R Guo C . Efficacy and safety comparison of small molecule anti-angiogenic drugs in the treatment of bone and soft tissue sarcomas: a network meta-analysis. BMC Cancer. (2025) 26:1–13. doi: 10.1186/s12885-025-15412-1 41372914 PMC12983772

[57] Dias ESD Pernomian LS Fernandes M Lenzi MC Gazzoni G Fonseca AC . Immune checkpoint inhibitors combined with tyrosine kinase inhibitors for soft-tissue sarcomas: a systematic review and single-arm meta-analysis. Oncologist. (2025) 30:1–9. doi: 10.1093/oncolo/oyaf337 41143677 PMC12578506

[58] Lai YS Wahyuningtyas R Aui SP Chang KT . Autocrine VEGF signalling on M2 macrophages regulates PD-L1 expression for immunomodulation of T cells. J Cell Mol Med. (2019) 23:1257–67. doi: 10.1111/jcmm.14027 30456891 PMC6349155

[59] Toulmonde M Guegan JP Spalato-Ceruso M Valentin T Bahleda R Peyraud F . Reshaping the tumor microenvironment of cold soft-tissue sarcomas with anti-angiogenics: a phase 2 trial of regorafenib combined with avelumab. Signal Transduction Targeted Ther. (2025) 10:202. doi: 10.1038/s41392-025-02278-9 40579407 PMC12205094

[60] Zheng B Ren T Huang Y Guo W . Apatinib inhibits migration and invasion as well as PD-L1 expression in osteosarcoma by targeting STAT3. Biochem Biophys Res Commun. (2018) 495:1695–701. doi: 10.1016/j.bbrc.2017.12.032 29225166

[61] Duan XL Guo JP Li F Xiu C Wang H . Sunitinib inhibits PD-L1 expression in osteosarcoma by targeting STAT3 and remodels the immune system in tumor-bearing mice. Future Oncol. (2020) 16:1815–24. doi: 10.2217/fon-2019-0725 32511016

[62] Ishikura N Sugimoto M Yorozu K Kurasawa M Kondoh O . Anti-VEGF antibody triggers the effect of anti-PD-L1 antibody in PD-L1(low) and immune desert-like mouse tumors. Oncol Rep. (2022) 47:1–10. doi: 10.3892/or.2021.8247 34958105 PMC8717122

[63] Benmebarek MR Oguz C Seifert M Ruf B Myojin Y Bauer KC . Anti-vascular endothelial growth factor treatment potentiates immune checkpoint blockade through a BAFF- and IL-12-dependent reprogramming of the TME. Immunity. (2025) 58:926–45:e10. doi: 10.1016/j.immuni.2025.02.017 40088889 PMC11981852

[64] Liu T Guan Y Bai L Ni B Zhang HY Zhang Y . Clinical study of adebrelimab in combination with apatinib and irinotecan for PD-1 inhibitor-ineffective advanced-stage gastric cancer: study protocol for a single-arm, single-centre, exploratory trial. BMJ Open. (2025) 15:e089286. doi: 10.1136/bmjopen-2024-089286 40467306 PMC12142080

[65] Wang J Zhou C Yao W Wang Q Min X Chen G . Adebrelimab or placebo plus carboplatin and etoposide as first-line treatment for extensive-stage small-cell lung cancer (CAPSTONE-1): a multicentre, randomised, double-blind, placebo-controlled, phase 3 trial. Lancet Oncol. (2022) 23:739–47. doi: 10.1016/s1470-2045(22)00224-8 35576956

[66] Yang H Yang L Liu Y Wang L . Adebrelimab in small cell lung cancer: from current advances to emerging combination strategy and challenge. Biologics: Targets Ther. (2025) 19:365–77. doi: 10.2147/btt.s500470 40475251 PMC12138384

[67] Wang BC Fu C Lin GH . The efficacy of adebrelimab compared with durvalumab and atezolizumab in untreated extensive-stage small-cell lung cancer: a survival analysis of reconstructed patient-level data. Front Immunol. (2023) 14:1185577. doi: 10.3389/fimmu.2023.1185577 37215120 PMC10196127

