# Supplementary figures and images for: Progression-free survival 3 of 22 months achieved through third-line therapy with adebrelimab in patient with recurrent chordoma: a case report
Source: Front Oncol. 2026 Jun 29;16:1850680. doi: 10.3389/fonc.2026.1850680 (PMC13357196; doi:10.3389/fonc.2026.1850680)

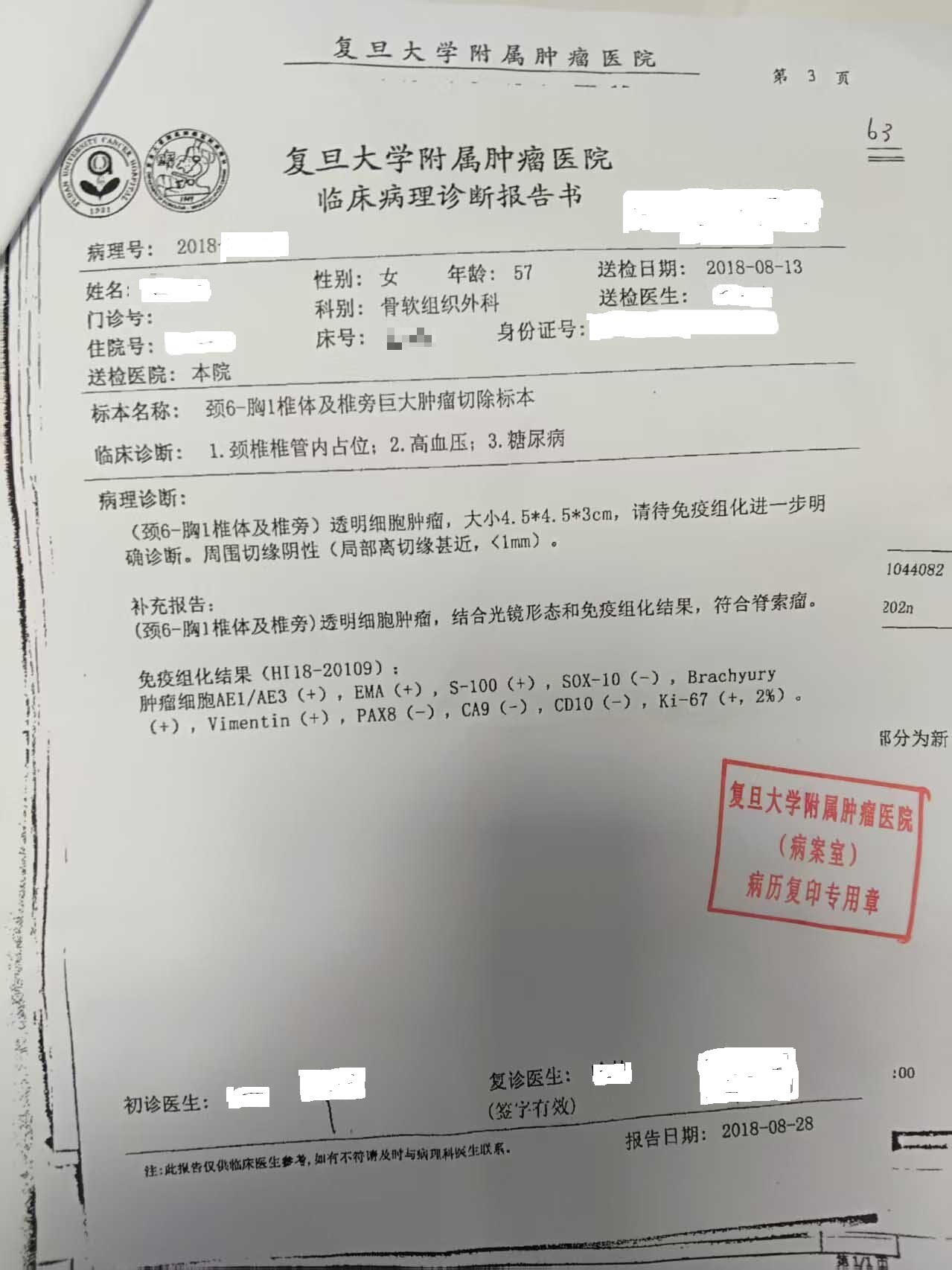

Supplement: Supplementary Figure S1 — Postoperative pathological report. [file Image1.jpg]
