# Supplementary material for: Progression-free survival 3 of 22 months achieved through third-line therapy with adebrelimab in patient with recurrent chordoma: a case report
Source: Front Oncol. 2026 Jun 29;16:1850680. doi: 10.3389/fonc.2026.1850680 (PMC13357196; doi:10.3389/fonc.2026.1850680)
Supplement: Supplementary Table S1 — Changes in laboratory test parameters during second-line and third-line therapies. [file Table1.docx]

Table S1. Changes in laboratory test parameters during second-line and third-line therapies

|  | Days* | WBC | HB | PLT | TT3 | FT3 | TT4 | FT4 | cholinesterase | albumin | CYFRA21-1 | NSE |
| --- | --- | --- | --- | --- | --- | --- | --- | --- | --- | --- | --- | --- |
| Unit |  | 10^9^/L | g/L | 10^9^/L | nmol/L | pmol/L | nmol/L | pmol/L | U/L | g/L | ng/ml | ng/ml |
| Reference range |  | 3.5-9.5 | 115-150 | 125-350 | 1.01-2.48 | 3.29-6.47 | 69.97-152.52 | 7.64-16.03 | 5300-11300 | 40.0-55.0 | 0-7.00 | 0-6.00 |
| Jun 24, 2022 | 1411 | 8.1 | 115 | 396 | 1.3 | 4.86 | 165.72 | 13.93 | 6996 | 37.7 | 0.94 | 3.48 |
| Sep 4, 2022 | 1483 | 7.8 | 107 | 386 |  |  |  |  |  | 35.4 | 2.04 | 2.79 |
| Sep 18, 2023 | 1862 | 8.04 | 109 | 351 | 0.81 | 4.4 | 121.19 | 11.83 | 6271 | 35.8 | 3.99 | 4.07 |
| Apr 1, 2024 | 2058 | 5.86 | 105 | 415 | 1.19 | 4.51 | 193.74 | 14.2 | 5646 | 33.7 | 35.17 | 4.38 |
| Apr 29, 2024 | 2086 | 5.95 | 111 | 270 | 1 | 5.17 | 164.57 | 11.88 | 7003 | 35.3 | 2.91 | 3.96 |
| Jun 7, 2024 | 2125 | 6.99 | 94 | 437 | 0.74 | 3.92 | 201.43 | 15.72 | 5684 | 31.1 | 6.06 | 4.35 |
| Oct 1, 2024 | 2241 | 12.42 | 70 | 404 | 0.34 | 4.91 | 154.07 | 4.91 | 3922 | 26.3 | 2.07 | 4.07 |
| Jan 23, 2025 | 2355 | 6.87 | 93 | 335 | 0.69 | 4.09 | 169.63 | 16.35 | 4884 | 30.5 | 12.35 | 14.47 |
| Apr 28, 2025 | 2450 | 8.1 | 79 | 582 | 0.4 | 3.04 | 147.2 | 13.29 | 4112 | 27.4 | 1.9 | 3.13 |
| Aug 12, 2025 | 2556 | 7.32 | 87 | 376 | 0.78 | 3.49 | 152.02 | 13.46 | 4561 | 29.6 | 3.42 | 9.13 |

*August 13, 2018 is designated as the reference date (Day 0).

Table S2. Efficacy assessment based on RECIST 1.1 criteria in second-line and third-line treatments

| Treatment | Second-line therapy | | | | Third-line therapy | | | | | | |
| --- | --- | --- | --- | --- | --- | --- | --- | --- | --- | --- | --- |
| Date | Jun 30, 2022 | Sep 9, 2022 | Sep 19, 2023 | Apr 4, 2024 | Jun 11, 2024 | Oct 8, 2024 | Jan 23, 2025 | May 6, 2025 | Aug 18, 2025 | Dec 3, 2025 | Feb 24, 2026 |
| Sum of longest diameters in target lesions (mm) | 131* | 105 | 90 | 132* | 112 | 119 | 118 | 118 | 130 | 141 | 153 |
| Efficacy evaluation | - | SD | PR | PD | SD | SD | SD | SD | SD | SD | SD |

*Baseline sum of longest diameters in target lesions for efficacy assessment.
